# Infliximab therapy in refractory sarcoidosis: a multicenter real-world analysis

**DOI:** 10.1186/s12931-022-01971-5

**Published:** 2022-03-09

**Authors:** Abdullah Sakkat, Gerard Cox, Nader Khalidi, Maggie Larche, Karen Beattie, Elisabetta A. Renzoni, Nilesh Morar, Vasilis Kouranos, Martin Kolb, Nathan Hambly

**Affiliations:** 1 Department of Medicine, King Abdulaziz University, Jeddah, Saudi Arabia; 2 Department of Medicine, McMaster University, Hamilton, Ontario Canada; 3 Firestone Institute for Respiratory Health, St Joseph’s Healthcare, Hamilton, Ontario Canada; 4 Division of Rheumatology, McMaster University, Hamilton, Ontario Canada; 5 Interstitial Lung Disease Unit, Royal Brompton Hospital, London, UK; 6 Department of Dermatology, Chelsea and Westminster Hospital, London, UK

**Keywords:** Sarcoidosis, Infliximab, Anti-TNF-α

## Abstract

**Background:**

Infliximab is a monoclonal antibody that binds and neutralizes circulating tumor necrosis factor-alpha, a key inflammatory cytokine in the pathophysiology of sarcoidosis. Despite the paucity of randomized clinical trials, infliximab is often considered a therapeutic option for refractory disease. Our study aimed to investigate the effectiveness of infliximab in patients with refractory sarcoidosis.

**Methods:**

Sarcoidosis patients from three tertiary centres were retrospectively identified by pharmacy records based on treatment with infliximab. Treatment with Infliximab was initiated in patients who failed first and second line immunomodulators as determined by a multidisciplinary team of Respirologists, Dermatologists, ENT specialists, Rheumatologists, and Neurologists. Participants were characterized by the primary organ for which infliximab was initiated and the total number of organs involved. Clinical outcomes were categorized as treatment success versus failure. We defined treatment success as (A) improvement of cutaneous, upper airway, lymph node, gastrointestinal, eye, or joint manifestations; or (B) improvement or no change in central nervous system (CNS) or pulmonary manifestations.

**Results:**

33 patients with refractory sarcoidosis were identified. The proportion of treatment success was 100% (95% CI 54.1–100) in CNS, 91.7% (95% CI 61.5–99.8) in cutaneous, 78.6% (95% CI 49.2–95.3) in pulmonary and 71.5% (95% CI 29.0–96.3) in upper airway disease. The use of infliximab was associated with a reduction prednisone dose by 50%.

**Conclusion:**

Infliximab is possibly an effective therapy for refractory sarcoidosis, with the greatest value in neurologic and cutaneous manifestations. Across all disease presentations, infliximab facilitated a clinically relevant reduction in corticosteroid dose. Relapse is common after discontinuation of infliximab.

## Background

Sarcoidosis is a multisystem disease of unknown etiology characterized pathologically by non-caseating granulomatous inflammation. Clinical manifestations are a consequence of such inflammatory change in target organs, with the lung being the most commonly affected (90% of cases) [1]. Sarcoidosis is a heterogeneous disease with a highly variable clinical course. Spontaneous remission is seen in about two-thirds of patients; however, 10–30% will have a chronic progressive disease [1, 2].

To date, there is no cure for sarcoidosis. Currently, available immunomodulatory therapies target granulomatous inflammation in a non-specific manner with the therapeutic goal of preventing irreversible end-organ damage [2]. If systemic treatment is indicated, first-line therapy includes oral corticosteroids, although potential significant adverse events hinder their long-term use [1–4]. Multiple second-line agents, including methotrexate, azathioprine, mycophenolate, hydroxychloroquine, and leflunomide, have been retrospectively studied in sarcoidosis [2, 4]. Indications for their use include an expected prolonged duration of therapy, presence of serious side effects related to corticosteroid therapy, or corticosteroid-resistant refractory disease—the definition of which has not been universally established [4].

Tumour necrosis factor-alpha (TNF-α) plays an essential role in the pathophysiology of sarcoidosis [5]. Infliximab is a humanized chimeric monoclonal antibody that binds and neutralizes circulating TNF-α. Despite the paucity of robust large randomized clinical trials (RCTs), infliximab is often considered a therapeutic option in the subgroup of patients with refractory disease or patients who are intolerant to first and second-line agents [4, 6]. Our study aimed to investigate the long-term effectiveness of infliximab in an international multi-centre retrospective cohort of patients with refractory sarcoidosis.

## Methods

Between Feb 2009 and May 2019, pharmacy prescription databases at three tertiary referral centres, (1) Firestone Institute of Respiratory health (FIRH), Hamilton, Ontario, Canada, (2) Royal Brompton Hospital, Chelsea, United Kingdom and (3) Westminster Hospital, London, United Kingdom were retrospectively reviewed to identify patients who were prescribed infliximab for the treatment of sarcoidosis. The confidentiality of patients’ data was maintained during the study. The study was approved by the research ethics board at each institution.

Patient charts were retrospectively reviewed. Clinical, physiologic, and radiographic data were collected using a standardized form to assess the course of sarcoidosis, both prior to, and after treatment with infliximab. The total dose of infliximab, duration of treatment, adverse events, and receipt of immunomodulators used prior to and concurrently with infliximab were documented. The reasons for treatment discontinuation were also collected.

Participants were characterized based on the primary target organ for which infliximab was initiated and the total number of systemic organs involved. Clinical outcomes were categorized as treatment success versus treatment failure. Treatment “success” was defined as: (a) improvement of cutaneous, upper airway, peripheral lymph node, gastrointestinal (GI), eye, or joint manifestations; or (b) improvement or no change in central nervous system (CNS) or pulmonary manifestations. Response to treatment was assessed based on objective measures whenever possible (Table 1).

**Table 1:** Assessment of treatment response

Organ/Disease manifestation	Assessment method	Definition of treatment success
Lung	Spirometry	Increase in absolute FVC or FEV1 by > 10% or No change in FVC or FEV1 (± 10% from baseline)
Skin	Clinical images	50% improvement in skin lesions in comparison to baseline images
Upper airway	Nasolaryngoscopy & CT	Improvement in structural change on serial exam and imaging
CNS	MRI	Improvement or no progression from baseline imaging
Peripheral lymph nodes	Clinical assessment	Resolution of lymphadenopathy
GI	Clinical assessment and laboratory	Resolution of symptoms and normalization of laboratory testing
Eye	Clinical assessment and eye exam	Resolution of symptoms and improvement of abnormalities on serial eye exam
Joint	Clinical assessment and laboratory	Resolution of symptoms and normalization of laboratory testing

*FVC* forced vital capacity; *FEV1* forced expiratory volume in one second; *CT* computed tomography; *MRI* magnetic resonance imaging; *CNS* central nervous system; *GI* gastrointestinal

Patients with latent tuberculosis (TB) were excluded from treatment using a TB skin test or interferon-gamma release assay (IGRA). Infliximab was initiated in patients who failed first and second line immunomodulators as determined by a multidisciplinary team of Respirologists, Dermatologists, ENT specialists, Rheumatologists, and Neurologists at the participating sites.

Patients received infliximab in both inpatient and outpatient settings, depending on institutional policy. The induction regimen consisted of 3–5 mg/kg dose at 0, 2, and 6 weeks. After this, infliximab was administered every 4–8 weeks. The total duration of therapy was individualized based on clinical response, adverse events, and the availability of payee funding. Follow-up and repeat testing frequency varied among patients due to variability in physician practice pattern and was tailored to the primary (index) organ(s) for which infliximab was initiated.

Descriptive statistics were expressed as frequency and percentage for categorical variables and as mean ± standard deviation (SD) for continuous variables. In patients with treatment success, the probability of clinical relapse following the discontinuation of infliximab was estimated by the Kaplan–Meier method. Statistical analysis was performed using R Project for Statistical Computing version 3.6.1.

## Results

The study population included 33 patients with biopsy-proven refractory sarcoidosis treated with infliximab. Baseline patient characteristics are shown in (Table 2). Before initiation of infliximab, all patients had previously been treated with corticosteroids and received at least one second-line immunomodulator; almost half of the patients (45%) had been treated with two second-line immunomodulators.

**Table 2:** Baseline patient characteristics

Number of patients	33
Age at diagnosis, (range)	51.6 (33–80)
Female, n (%)	23 (70)
Race, n (%)	
White	20 (61)
Black	11 (33)
South Asian	1 (3)
Middle Eastern	1 (3)
Non-smoker, n (%)	17 (51.5)
Negative TB skin test, n (%)	31 (94)
Negative IGRA, n (%)	2 (6)
Histologically proven sarcoidosis, n (%)	33 (100)
Pulmonary function testing*	
FVC, % predicted	83.4 ± 28
Range	44.8–135.6
FEV1, % predicted	73.5 ± 28.6
Range	23.0–121.8
DLCO, % predicted	57.7 ± 24.9
Range	24.0–99.7
Immunosuppressive therapy before initiation of infliximab, n (%)**	
Methotrexate	27 (82)
Azathioprine	13 (39)
Hydroxychloroquine	20 (61)
Leflunomide	1 (3)
Mycophenolate	5 (15)
IV methylprednisolone	10 (30)
Bronchial steroid injection	1 (3)
Cyclophosphamide	4 (12)
Rituximab	1 (3)

*IGRA* Interferon-Gamma Release Assays; *FVC* forced vital capacity; *FEV1* forced expiratory volume in one second; *DLCO* diffusing capacity of the lung for carbon monoxide

*PFT or spirometry data available for only 30 patients

**All patients were treated with oral corticosteroid prior to initiation of infliximab

Disease characteristics, index organ for which infliximab was initiated and concomitant therapies are summarized in (Table 3). More than half of patients (64%) had ≥ 2 organs involved with the most commonly affected being the lung (n = 30, 91%) and skin (n = 15, 45%). The most common indications for infliximab were pulmonary (n = 14, 42%) and cutaneous disease (n = 12, 28%). During treatment with infliximab, one-third of patients were treated with an additional immunomodulator, while over half of patients (61%) required treatment with both corticosteroids and a second-line immunomodulator during their treatment course.

**Table 3:** Granulomatous organ involvement

	Number of patient (%)
Organ involvement	
Lung	30 (91)
Skin	15 (45)
Lupus pernio	8
Other skin manifestation	7
Upper airway	9 (27)
Eye	7 (21)
Liver	4 (12)
CNS	6 (18)
Cardiac	3 (9)
GI	1 (3)
Spleen	3 (9)
Peripheral lymph node	2 (6)
Bone marrow	1 (3)
Muscle	1 (3)
Arthritis	1 (3)
Organ involvement	
1	12 (36)
2	7 (21)
≥ 3	14 (43)
Multiorgan (≥ 2)	21 (64)
Organ/manifestation for which infliximab was initiated	
Lungs	14 (33)
Skin	12 (28)
Upper airway	7 (16)
CNS	6 (14)
Peripheral lymph node	1 (2)
GI	1 (2)
Uveitis	1 (2)
Arthritis	1 (2)
Concomitant therapy	
Corticosteroid alone	5 (15)
Second line immunosuppressive alone	9 (27)
Corticosteroid + second line immunosuppressive	19 (58)

*GI* gastrointestinal; *CNS* central nervous system

**Fig. 1:**
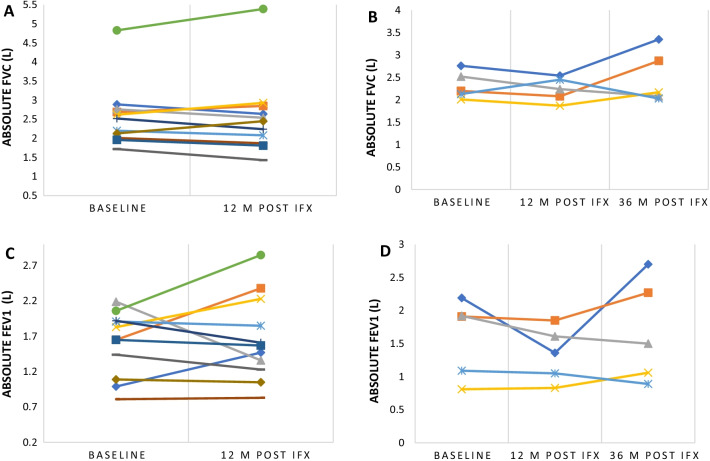
**A** Individual patient (n = 11) absolute FCV at baseline and at 12 months post treatment with infliximab. **B** Individual patient (n = 5) absolute FCV at baseline, 12 and 36-months post treatment with infliximab. **C** Individual patient (n = 11) absolute FEV1 at baseline and at 12 months post treatment with infliximab. **D** Individual patient (n = 5) absolute FEV1 at baseline, 12 and 36-months post treatment with infliximab. *IFX* infliximab; *FVC* forced vital capacity; *M* month; *L* liters

**Fig. 2:**
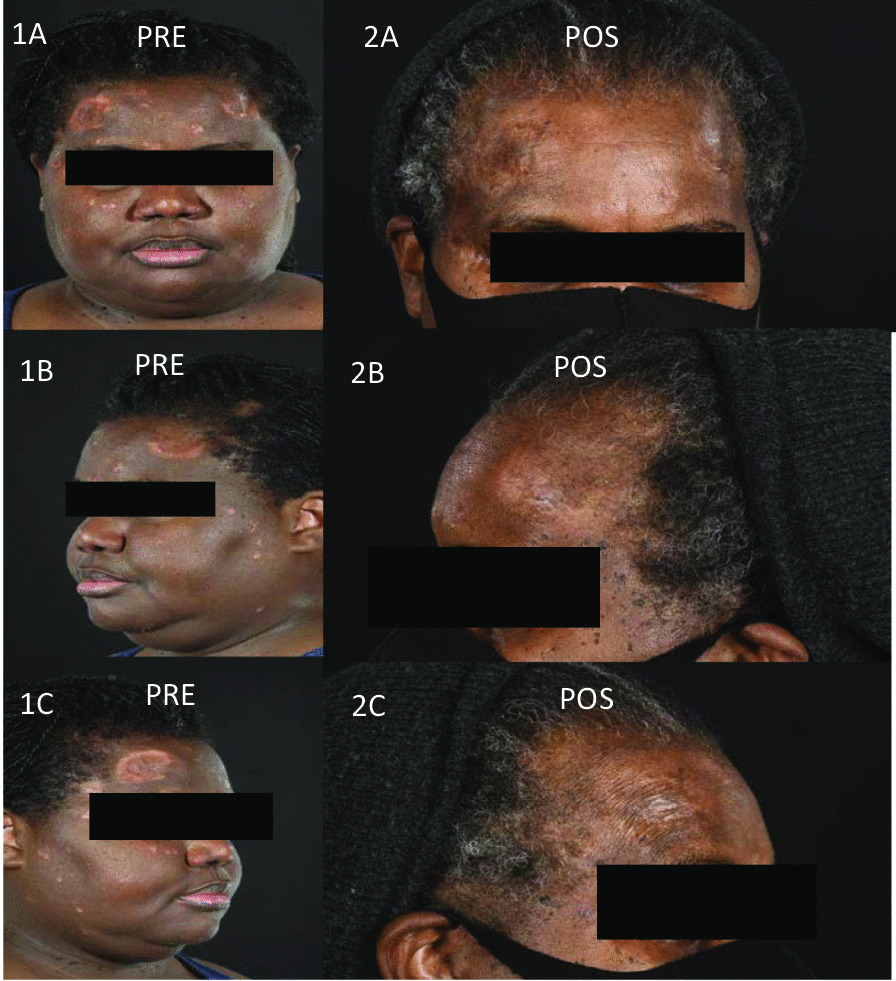
Pre-treatment images of a patient with cutaneous sarcoidosis presenting with annular lesions, papules and plaques resulting in scarring, atrophy, hyperpigmentation and hypopigmentation (**1A**–**1C**). Post treatment images shows healed cutaneous sarcoidosis lesions with post-inflammatory hypopigmentation and atrophy (**2A**–**2C**)

The proportion of treatment success was 100% (95% CI 54.1–100) in CNS, 91.7% (95% CI 61.5–99.8) in cutaneous, 78.6% (95% CI 49.2–95.3) in pulmonary and 71.5% (95% CI 29.0–96.3) in upper airway disease (Table 4). In patients who were treated with infliximab for lung disease (n = 14), serial spirometry data were available for 11 patients. Individual patient FVC at baseline and after treatment with infliximab are displayed in (Fig. 1). Overall, FVC and FEV1 remained stable over time. Twelve months after treatment with infliximab, the absolute increase in FEV1 was + 90 ml (55%) [95% CI − 0.31 to 0.39] that was associated with a negligible change in FVC − 20 ml (− 0.77%) [95% CI − 0.18 to 0.24]. This effect continued to be seen in a subgroup of patients (n = 5) who completed 36 months of treatment. Figure 2 shows clinical images of a patient with cutaneous sarcoidosis before and after treatment with infliximab. Treatment success was also observed in single cases of sarcoidosis related uveitis, GI involvement, painful peripheral lymphadenopathy, and arthritis.

**Table 4:** Clinical outcomes following initiation of infliximab

Organ/manifestation for which infliximab was initiated	Number of patients	Treatment success % (95% C.I)
Lung	14	78.6% (49.2–95.3)
Cutaneous	12	91.7% (61.5–99.8)
Upper airway	7	71.5% (29.0–96.3)
CNS	6	100% (54.1–100)
Peripheral lymph node	1	100% (2.5–100)
Gastrointestinal	1	100% (2.5–100)
Uveitis	1	100% (2.5–100)
Arthritis	1	0% (0.0–97.5)
Total	43	83.7% (69.3–93.2)

**Fig. 3:**
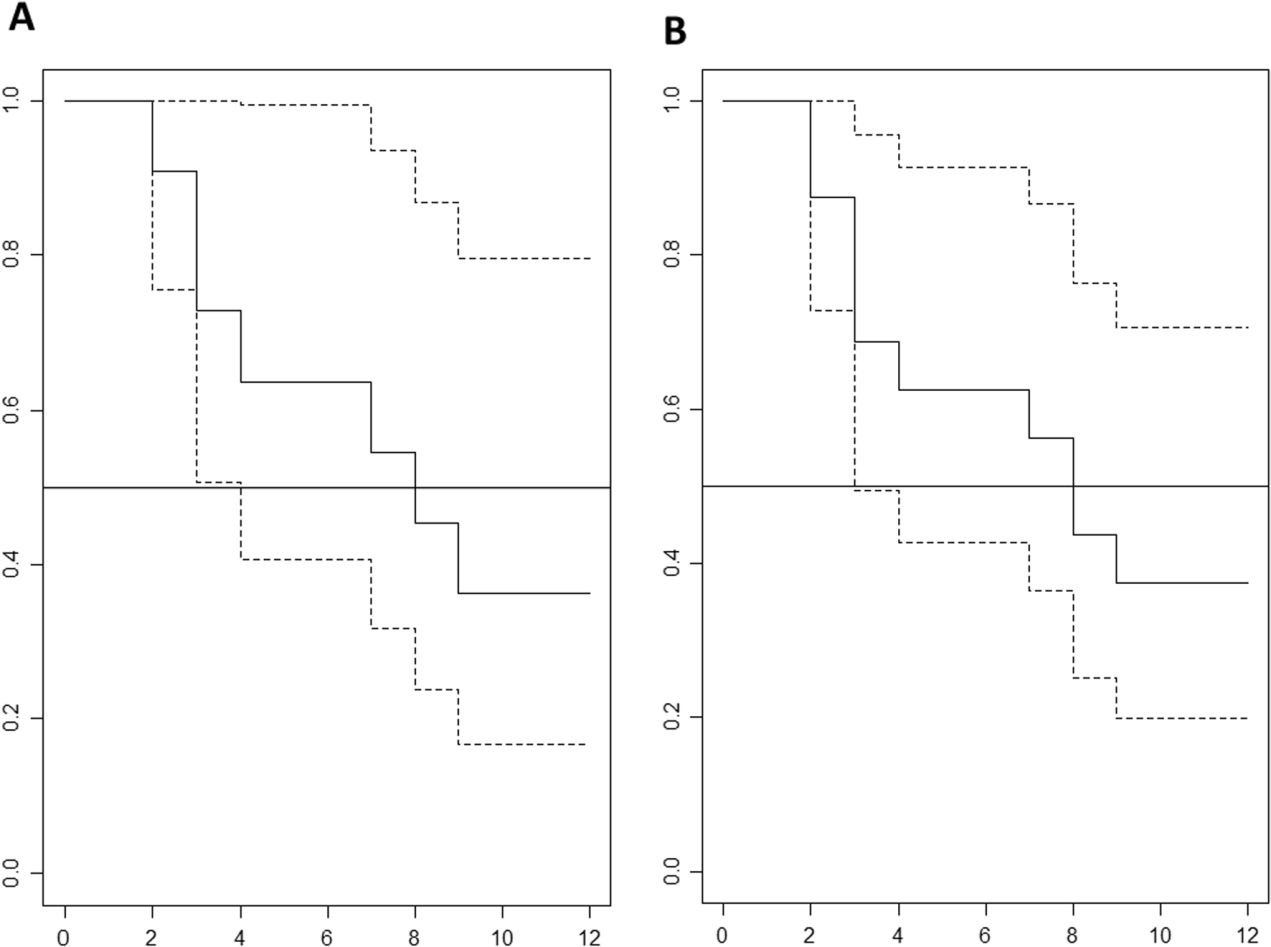
Kaplan–Meier analysis of time (months) to clinical relapse after discontinuation of infliximab. Panel **A** demonstrates the time to clinical relapse of index organ in individual patients who initially responded to infliximab. Panel **B** describes the time to clinical worsening in index organs for which infliximab was prescribed

Data on prednisone dose before and after treatment with infliximab were available for 22 patients. The use of infliximab was associated with a reduction in the mean prednisone dose by 50% from baseline mean of 21.7 ± 12.7 mg/day to 10.5 ± 8.3 mg/day as recorded on the date of last follow up. Patients who discontinued infliximab following either an improvement or resolution of disease activity were followed for a mean of 36.9 (5–82) months. At the time of study completion, 7/11 patients (63.6%), and 10/16 index organs (62.5%), relapsed following treatment discontinuation. Kaplan–Meier analysis revealed a median time to relapse after discontinuing infliximab of 8 ± 2.04 months for individual patients and 8 ± 2.55 months for index organ for which infliximab was prescribed (Fig. 3).

Adverse events related to infliximab were documented in 17 patients (51%), details of which are summarized in (Table 5). The most commonly reported adverse events included non-serious infections which occurred in 9/33 patients (27%). Permanent treatment discontinuation due to adverse events occurred in 7/33 patients (21%). Reasons for permanent discontinuation included: (a) two cases of recurrent infusion reactions associated with pruritus and paraesthesias; (b) four episodes of anaphylaxis, three of which occurred following a period of treatment interruption; and (c) an acute flare of lupus pernio in one patient, following her first infliximab infusion. One patient died of progressive respiratory failure in the setting of severe fibrotic lung disease. The decline in lung function was deemed secondary to progressive fibrosing interstitial lung disease rather than a complication of infliximab therapy.

**Table 5:** Adverse events

Adverse event	Number of patients (%)
None	8 (24)
Pneumonia	6 (18)
Leukopenia	5 (15)
Infusion reaction	4 (12)
Minor infection*	3 (9)
Paraesthesias	2 (6)
Anaphylaxis	4 (12)
Flare of cutaneous disease	1 (3)
Chest pain	1 (3)
Headache	1 (3)
Asthma	1 (3)
Permanent discontinuation due to adverse event	7 (21)

*One case of cellulitis, cholecystitis, and recurrent sinusitis

## Discussion

Our multicentre retrospective study reports real-world data of our cumulative experience in treating patients with various manifestations of severe sarcoidosis refractory to first and second line immunomodulatory therapies. The results of our evaluation suggest that infliximab is an apparent effective therapy in the setting of refractory sarcoidosis, which permits a clinically relevant reduction in corticosteroid dose, and although adverse events are common permanent discontinuation was not required in the majority of patients. We also observed that index organ relapse was frequently appreciated within a year of infliximab discontinuation.

Sarcoidosis is a heterogeneous disease with expected variable outcomes and response to treatment [1]. In our study, patients were stratified according to the index organ for which infliximab was initiated. The decision to initiate infliximab was carried out by a team of specialists with expertise in managing sarcoidosis. Whilst treatment response was assessed by objective measures whenever possible, we acknowledge that many of the measures utilized have not been standardized or validated. The definition of treatment “success” in sarcoidosis is variable, and no standardized definitions or criteria have yet been established. In our study, we characterized treatment success based on the involved index organ. We defined treatment success as an improvement or resolution for all organs except CNS or pulmonary disease, where, in clinical practice, stabilization of disease with no further progression is often considered a therapeutic success.

Treatment of CNS sarcoidosis is almost always warranted, as progressive disease can lead to irreversible disability. Infliximab is currently considered the treatment of choice in patients with CNS sarcoidosis for whom treatment with systemic corticosteroids and second-line immunomodulatory therapies failed [6, 7]. In our study, infliximab demonstrated a high level of effectiveness in treating CNS sarcoidosis, similar to previously published case series and observational studies [8–10].

Patients with cutaneous disease demonstrated an excellent response to infliximab. Only one patient with cutaneous sarcoidosis failed treatment due to a paradoxical reaction with worsening lupus pernio and nodular disease after infliximab infusion. This is a rare adverse event of infliximab and has been described in the literature [11].

Upper airway involvement is a rare manifestation of sarcoidosis with an incidence rate of approximately 5% [12]. In our cohort, two-thirds of the patients with upper airway sarcoidosis also had skin involvement. This association has been previously described [13, 14]. The role of infliximab in upper airway disease has not been well established; this could be due to the rare occurrence and/or responsiveness to first and second line immunomodulatory therapies. Our results suggest that the use of infliximab was associated with successful treatment response in more than two-thirds of patients.

Although RCT data in the field of sarcoidosis is scarce, one of the few double-blind, placebo-controlled RCTs in this field, evaluating the use of infliximab in the setting of chronic pulmonary sarcoidosis, demonstrated an improvement in FVC by 2.5% predicted at 24 weeks. This trial was criticized due to the observed small improvement in FVC and potential lack of clinically meaningful outcomes [15]. Another prospective open-label trial of 34 patients with pulmonary sarcoidosis treated with infliximab showed an improvement in quality of life and FVC (6.64% predicted) [16]. Marginal improvement or stability was also seen in observational studies [17, 18]. In our cohort, the use of infliximab was associated with a favourable response. Although there was no demonstratable improvement in lung function, spirometric parameters remained similar to baseline value 12 months following initiation of therapy. This effect continued to be seen in patients who completed 36 months of treatment. In our opinion, stability should be considered a treatment success, as halting disease progression potentially prevents the development of end-stage lung disease and obviates the necessity for lung transplantation.

Single cases of sarcoidosis related uveitis, GI, painful peripheral lymphadenopathy and arthritis improved with infliximab. Due to the low number of individual cases, we could not elaborate further on the treatment effect of these index organs.

Corticosteroids are the cornerstone treatment of sarcoidosis and have been used for this indication for more than 50 years [4]. Unfortunately, their long-term use is associated with many undesired side effects that often outweigh their potential short-term benefits [1–4]. In our cohort, the use of infliximab was associated with a clinically relevant reduction in mean prednisone dosage (50%). This is a critically important outcome; although it does not directly impact the disease, it reduces the adverse effects of corticosteroids that potentiate pre-existent morbidity.

Relapse following discontinuation of infliximab was first reported by Panselinaa et al*.* who found a high rate of relapse (86%) after discontinuing infliximab [19]. Multiple observational studies have subsequently reported a relapse rate between 44 to 62% [9, 20, 21]. In our cohort, 10 of 16 (62.5%) index organs relapsed after treatment discontinuation. It is important to note that infliximab was discontinued in these scenarios following either an improvement or resolution of disease activity. Previous studies have demonstrated that high mediastinal standardized uptake value (SUV) max score on fluorodeoxyglucose (FDG)-positron emission tomography (PET) and high serum soluble interleukin-2 receptor concentration at the initiation of therapy are able to predict relapse after discontinuation of infliximab [20]. We were unable to assess predictive factors for relapse due to small sample size and the retrospective nature of our study. Experts suggest gradual prolongation of the interval between infliximab doses before discontinuation to decrease the risk of relapse [7]. Longer duration of treatment may be required to prevent relapse; however, this has not been studied and may be associated with increased risk of adverse events.

Adverse events related to infliximab are well recognized with multiple black box warning related to severe infection and risk of malignancy. The rate of adverse events in our cohort was consistent with previously published observational studies, with infection being the most common [18, 22]. An additional immunomodulator is typically prescribed while patients receive infliximab to prevent the formation of antibodies against the chimeric infliximab antibody. Meta analyses of RCTs and Food and Drug Administration (FDA) reports showed no difference in the risk of serious infection between TNF-α inhibitor monotherapy versus combination therapy with immunomodulators and/or systemic corticosteroid, although observational studies have reported an increased risk of infection with combination therapy [23–26]. In our cohort, the rate of infection was similar whether infliximab was added to a single immunomodulator or was added to a combination of corticosteroids and immunomodulatory therapy. It is noteworthy to mention that our observational study was not designed to investigate the safety of infliximab; hence, the reported adverse events should only be interpreted as incidental findings in the context of the administration of infliximab.

Limitations of this study include small sample size, lack of comparison-placebo group, and potential bias inherited by the retrospective study design.

Due to the lack of robust RCT evidence, infliximab is currently used “off-label” to treat advanced sarcoidosis. Most government-based and private payer systems do not cover the expense of treatment. However, the dramatically heterogeneous nature of sarcoidosis, the progressive nature of refractory disease, the limited efficacy and poor tolerability associated with the conventional “off-label” therapies (including corticosteroids), and the strength of existing cohort and experiential data suggest that waiting for prospective RCTs to approve such treatments in sarcoidosis is both unrealistic and potentially unethical.

## Conclusion

Infliximab is an apparent effective therapy for refractory sarcoidosis, with greatest value in managing the neurologic and cutaneous manifestations of the disease. Across all disease presentations, infliximab facilitated a clinically relevant reduction in corticosteroid dose. Unfortunately, relapse after discontinuation of infliximab is common. A standardized definition of refractory sarcoidosis and validated tools to assess treatment response are needed to harmonize future patient care and research.

## Data Availability

The datasets used and/or analysed during the current study are available from the corresponding author on reasonable request.
